# Effect of Improvement in Sarcopenia on Functional and Discharge Outcomes in Stroke Rehabilitation Patients

**DOI:** 10.3390/nu13072192

**Published:** 2021-06-25

**Authors:** Tatsuya Matsushita, Shinta Nishioka, Shiori Taguchi, Anna Yamanouchi, Yuka Okazaki, Kana Oishi, Ryusei Nakashima, Tatsuya Fujii, Yoshiharu Tokunaga, Shinya Onizuka

**Affiliations:** Nagasaki Rehabilitation Hospital, 4–11, Ginya–machi, Nagasaki 8500854, Japan; wkymf348@yahoo.co.jp (T.M.); ksharu1216@gmail.com (S.T.); s.y.k.o@outlook.jp (A.Y.); okazaki@zeshinkai.or.jp (Y.O.); dh-reha@zeshinkai.or.jp (K.O.); r-nakashima@zeshinkai.or.jp (R.N.); fujii@zeshinkai.or.jp (T.F.); tokunaga@zeshinkai.or.jp (Y.T.); onizuka@zeshinkai.or.jp (S.O.)

**Keywords:** activities of daily living, rehabilitation, sarcopenia, stroke

## Abstract

This cross-sectional study investigated the proportion of patients’ recovery from sarcopenia status and the relationship between improvement in sarcopenia (IS) and function and discharge outcome in hospitalized patients with stroke. This study included patients with stroke, aged 65 years or more, with a diagnosis of sarcopenia, who were admitted to a convalescent rehabilitation ward. Sarcopenia was diagnosed according to the Asian Working Group for Sarcopenia 2019 criteria. Patients were divided according to the presence or absence of sarcopenia at discharge: IS group and non-improvement in sarcopenia (NIS) group. Among the 227 participants (mean age: 80.5 years; 125 females), 30% (69/227) of the patients were in the IS group, while 70% (158/227) were in the NIS group. The IS group showed a higher Functional Independence Measure (FIM) than the NIS group (median 112 vs. 101, *p* = 0.003). The results demonstrated that IS was independently associated with higher FIM (partial regression coefficient, 5.378; 95% confidence interval (CI), 0.709–10.047). The IS group had higher odds of home discharge than the NIS group (odds ratio, 2.560; 95% CI, 0.912–7.170). In conclusion, recovery from sarcopenia may be associated with better function in patients with stroke.

## 1. Introduction

Sarcopenia, a progressive and systemic skeletal muscle disorder, increases the risk of adverse clinical outcomes, including falls, fractures [1,2], physical disability [3] and poor quality of life [4]. Sarcopenia is a syndrome that can be divided into age-related, disease-related, inactivity-related and poor nutrition-related forms [5]. The etiology of this syndrome is multifactorial, with the acute and chronic diseases playing a key role in promoting sarcopenia. In fact, it is well known that sarcopenia is prevalent in patients with cardiovascular disease (31.4%), dementia (26.4%) and diabetes (31.1%) [6].

Sarcopenia that occurs in patients after a stroke is specifically referred as “stroke-related sarcopenia” [7]. In recent years, more attention has been given to research about the modifications in the characteristics of the muscle tissue after a stroke [8]. In this context, stroke-related sarcopenia is characterized by a rapid decline in muscle mass after onset, muscle fiber-type shifts, determining bilateral differences in the physical and functional performance of brain lesions, impaired feeding and complex systemic metabolic changes [7,9]. Stroke-related sarcopenia has been described in a variety of settings, with a prevalence of 16.8% in the community [10] and 48.3–60.3% in the convalescent rehabilitation ward [11,12]. The prevalence of stroke is expected to increase in the future, and in parallel, the incidence of stroke-related sarcopenia is also expected to increase [10,13,14]. Stroke-related sarcopenia can exacerbate patients’ ability to perform activities of daily living (ADL). Indeed, our previous study demonstrated that sarcopenia is a predictor of poor ADL in patients after stroke [12]. In addition, the presence of sarcopenia in patients with stroke is associated with lower ADL, less overall improvement in patients with dysphagia and decreased likelihood of hospital discharge [15].

Early and appropriate multimodal interventions are required to recover from a sarcopenic status [5]. For patients with stroke, combined therapy of rehabilitation and nutrition is recommended in the guidelines for ADL improvement [16]. Thus, several randomized controlled trials have been conducted with post-stroke patients with sarcopenia in a convalescent rehabilitation setting [17,18]. The studies showed significant increases in muscle mass in the intervention group compared to the control group. However, whether the patients recovered from sarcopenia was not discussed in these studies. In this context, in the case of recovering from sarcopenia with a corresponding improvement in muscle mass and/or muscle strength over the cutoff values defined by the diagnostic criteria for sarcopenia, it is expected to have a more significant effect on the recovery of ADL capability in patients with stroke. To the best of our knowledge, no study has shown how the natural course of stroke rehabilitation modifies the sarcopenic status. Investigation of the modification in sarcopenic status will be beneficial as it will provide fundamental information for establishing intermediate outcomes in the future intervention studies.

Therefore, the present study is a cross-sectional study that aims to investigate the changes in sarcopenic status among stroke patients in convalescent rehabilitation wards, and whether these changes are associated with functional and discharge outcomes in these patients.

## 2. Materials and Methods

### 2.1. Study Participants and Design

A cross-sectional study was conducted involving consecutive patients after stroke who were admitted to and discharged from a single convalescent rehabilitation hospital in Japan, between January 2017 and October 2020. Convalescent rehabilitation wards aim to maximize the recovery of ADL capability, enabling patients to return to their own homes under a multidisciplinary rehabilitation program provided by a team that involves medical doctors, nurses, care workers, physical therapists, occupational therapists, speech–language–hearing therapists, social workers, registered dietitians, dental hygienists and also pharmacists [19]. The medical costs of the wards for patients with stroke are covered during the first 180 days by public health insurance. The rehabilitation program is composed of physical therapy (PT), occupational therapy (OT) and speech–language–hearing therapy (ST) and is provided for up to 180 min/day, aiming to fit the patient’s disability and functional ability. PT includes range of motion exercises, paralyzed limb facilitation, basic movement training, gait training, ADL training and resistance training. OT comprises ADL training, instrumental ADL training such as cooking and shopping, environmental adjustments to improve movement and return-to-work training. ST consists of verbal communication training for aphasia and dysarthria as well as feeding and swallowing training.

The eligibility criteria for this study included patients aged 65 years or more with a diagnosis of sarcopenia at the time of admission to the hospital and for whom skeletal muscle mass index (SMI) and hand-grip strength (HG) measurements were available. The exclusion criteria included being transferred to an acute care hospital owing to an exacerbation of clinical conditions or surgery (e.g., recurrent stroke, severe pneumonia and surgical procedures such as gastrostomy) and missing data of outcome measures. Because orthopedic prostheses/implants may influence the results of bioelectrical impedance analysis [20], this study excluded patients with metallic implantation. In addition, this study excluded patients who elapsed more than 60 days from the onset to hospitalization and patients hospitalized for more than 180 days in convalescent rehabilitation wards because the upper dose limit of rehabilitation therapy for these patients was less than that of patients within limits for hospitalization (180 min/day vs. 120 min/day). Although this rule was abolished in March 2020, the same condition was applied to patients admitted between April 2020 and October 2020 to align the patients’ backgrounds.

### 2.2. Ethical Consideration

This study was conducted in full agreement with the Code of Ethics of World Medical Association (Declaration of Helsinki), being approved by the Ethics Committee of Nagasaki Rehabilitation Hospital (approval number: R2-09). The requirement for informed consent was waived by the committee because the patients were informed that their data had been collected through the clinical practice and could be used for any reason. In addition, these data were anonymized during the analysis. Instead, an opt-out option was provided to allow patients to withdraw from the database at any time.

### 2.3. Outcome Measures

The primary outcome established in this study was the Functional Independence Measure (FIM). FIM is widely internationally used as an assessment of ADL capability [21]. It consists of a total of 18 items (13 items in the motor domain and 5 items in the cognitive domain) on a 7-point ordinal scale, ranging from complete independence to total assistance. The motor domain is divided into four main categories, namely self-care, sphincter control, transfer and locomotion, and the cognitive domain is divided into two main categories, namely communication and social cognition. The FIM score ranges from 18 to 126 points. A higher FIM score is compatible with more independent patients. The secondary outcomes established in this study were FIM-motor, FIM-cognitive and proportion of home discharge. Experienced physical, occupational and speech–language–hearing therapists, nurses and care workers assessed the FIM score. In the previous study, the intraclass correlation value for inter-rater reliability of FIM was 0.95 [22].

### 2.4. Data Collection

In this study, the characteristics from the medical charts were collected, namely age, sex, stroke subtype (i.e., ischemic stroke, hemorrhagic stroke), comorbidities (the Charlson Comorbidity Index (CCI)) [23], days between onset and admission in convalescent rehabilitation wards, FIM score, FIM gain, pre-stroke care needs, lower limb motor paralysis, height, body weight, body mass index (BMI), nutrition route (e.g., oral intake, tube feeding), Malnutrition Universal Screening Tool (MUST) [24] score, energy intake, protein intake, length of stay, discharge outcome (home or others) and daily rehabilitation dose. FIM gain was calculated by subtracting FIM at admission from FIM at discharge. Pre-stroke care needs were determined by whether or not the patient was certified by the public long-term care insurance [12]. This is regulated under the public long-term care system in Japan and all citizens who have Japanese nationality have an obligation to manage the long-term care of the elderly as a whole. Lower limb motor paralysis was evaluated by physical therapists using the Brunnstrom recovery stage (BRS) [25]. Patients were then classified into three groups: BRS I to IV, BRS V to VI and absence [12,26]. MUST is a malnutrition screening tool, being evaluated on the day of admission by registered dietitians. Average energy and protein intake were calculated by registered dietitians as the mean during the first 3 days of hospitalization based on the medical record of visual estimation for food intake by a nursing staff [27]. Moreover, in the case of patients that received enteral and/or parenteral nutrition, the contained energy and protein were also taken into account.

### 2.5. Definition of Sarcopenia

All participants were Japanese. Diagnosing sarcopenia in Asian people requires some special considerations because of anthropometric and cultural or lifestyle-related differences (i.e., relatively smaller body size, higher adiposity and less mechanized/more physically active lifestyles) from Western populations [28]. We applied the Asian Working Group for Sarcopenia (AWGS) 2019 criteria in this study because we believe that it is better adapted to Asian populations than the European Working Group on Sarcopenia in Older People (EWGSOP2) criteria [5]. Therefore, patients with a low SMI plus low muscle strength were diagnosed with sarcopenia. This study did not use physical performance tests for sarcopenia diagnosis (e.g., chair standing test and walking speed test) because patients after stroke often have difficulty walking due to the hemiplegia. Cutoff values for sarcopenia diagnosis were SMI < 7.0 kg/m^2^ for men and <5.7 kg/m^2^ for women and HG < 28 kg for men and <18 kg for women. SMI was obtained using bioelectrical impedance analysis (BIA) (InBody S10; InBody Japan, Tokyo, Japan) and HG, which were both measured by a trained physical therapist and registered dietitian within 7 days after admission and also within 7 days before discharge. BIA was measured in patients in the supine position after 5 min of rest and more than 30 min after eating. HG was measured in the dominant hand (or, in the case of hemiplegia, in the nonparalyzed hand) with the patient seated and with the arm straight, using a Smedley hand dynamometer grip D (Takei Scientific Instruments, Niigata, Japan). HG was measured twice at the same time of day, and the higher result was recorded based on the AWGS 2019 criteria [28].

### 2.6. Sample Size Calculation

The sample size was calculated using EZR and required to achieve a statistical power of 0.8 and an alpha error of 0.05 [29]. We inferred that a difference of 22 in the FIM-total between groups was detected based on the minimum clinically important difference (MCID). MCID, which was proposed in 1989 [30], is a concept in a patient-reported outcome measure of the smallest change value that can be interpreted as beneficial for the patient. If the observed difference has reached MCID, it implies a clinically relevant difference regardless of statistical significance. In a prospective case series of 113 patients with stroke in a long-term acute care setting conducted over a 9-month period, Beninato et al. defined the MCID of FIM as 22 [31]. The MCID of FIM score is clinically relevant and can be used during research as an outcome measure. Studies have used these as outcomes in nutritional management for older patients with sarcopenic dysphasia and GABAergic drug use for patients after stroke [32,33]. We considered these values to be valid and useful, and we used them in the sample size calculation. FIM was normally distributed with a standard deviation (SD) of 32.7 [19]. Assuming an improved to non-improved sarcopenia group ratio of 1:1, it was estimated that each group needed a sample size of at least 35 patients.

### 2.7. Statistical Analysis

Patients who had recovered from sarcopenia at discharge were defined as belonging to the improvement in sarcopenia (IS) group, while patients who remained affected by sarcopenia were assigned to the non-improvement in sarcopenia (NIS) group.

Continuous variables were reported as mean and SD for parametric data, or as medians and 25th to 75th percentiles (interquartile ranges; IQR) for non-parametric data. We used *t*-test for normal distribution data, Mann–Whitney U tests for data with skewed distributions and chi-square tests for categorical variables to compare the two groups. A multiple linear regression analysis was used for FIM-total, FIM-motor and FIM-cognitive. The selected covariates to adjust for bias were, age, sex, days between stroke onset to admission, FIM at admission, pre-stroke care needs, HG, SMI, lower limb motor paralysis, stroke subtype, MUST, CCI, tube feeding, energy intake and protein intake. A logistic regression analysis was used to determine whether the IS was independently associated with return to home. As covariates, age, sex, pre-stroke care needs and FIM at admission were selected. Moreover, the covariates that were associated with the outcome in previous studies were also selected [12,15]. In addition, we performed multiple linear regression analysis for FIM-total, FIM-motor and FIM-cognitive using the improvement in HG instead of IS to distinguish the impact of natural recovery of muscle mass after stroke from “improvement in sarcopenia”. To minimize the effect of stroke itself, we excluded tetraplegic patients from this analysis so that all data on HG were measured on the non-paretic side. Similarly, binary logistic regression analysis for hospital discharge was performed. The threshold for significant was *p* < 0.05. All statistical analyses were conducted using EZR [29].

## 3. Results

**Figure 1:**
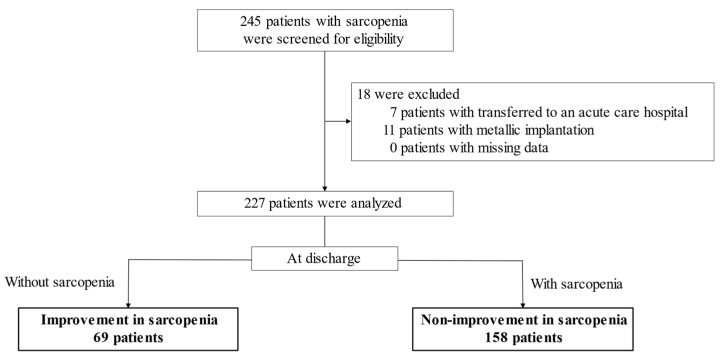
Flowchart of the studied participants.

From the 245 participants first enrolled in the study, those who met the exclusion criteria were then excluded (7 patients transferred to an acute care hospital and 11 patients with metallic implantation). As a result, a total of 227 eligible patients were included in this study and analyzed (mean age: 80.5 years; 102 males and 125 females) (Figure 1). The sarcopenic status of all participants was measured within 7 days of admission and within 7 days of discharge. Specifically, the median number of days that sarcopenic status was measured after admission was 2 in both the IS and NIS groups, (*p* = 0.850). The median number of days that sarcopenic status was measured before discharge was 3 in both the IS and NIS groups (*p* = 0.112).

Table 1 summarizes the characteristics of the 227 patients included in this study. From these, 72% (164/227) patients had experienced an ischemic stroke and 28% (63/227) had suffered a hemorrhagic stroke. Thirty percent (69/227) of the patients were in the IS group and 70% (158/227) were in the NIS group. The IS group was significantly younger (*p* = 0.002) and had higher FIM cognitive domain (*p* = 0.009), BMI (*p* = 0.019), SMI (*p* < 0.001) and HG (*p*-value < 0.001) than the individuals with NIS. The CCI score was significantly lower in the IS group (*p*-value = 0.017). The IS group had fewer patients that required care before the onset of stroke compared to the NIS group (10% vs. 30%, *p*-value < 0.001). At the time of discharge from the convalescent rehabilitation wards, compared to the time of admission, the patients’ muscle strength (HG) increased in 70% (159/227), while muscle mass (SMI) increased in 65% (148/227).

Table 2 shows the discharge outcomes of the groups. The IS group had significantly higher FIM-total (*p* = 0.003), FIM-motor (*p* = 0.009), FIM-cognitive (*p* = 0.001), FIM-total gain (*p* = 0.002), FIM-motor gain (*p* = 0.002), SMI (*p* < 0.001) and HG (*p* < 0.001). The IS group were significantly more likely to return home than the NIS group (91% vs. 77%, *p* = 0.015). The participants were offered a median rehabilitation program of 176 min/day that comprised a (median) PT of 78.9 min/day, OT of 55.3 min/day and ST of 40 min/day. The PT, OT and ST in the IS and NIS groups were 80.2 min/day vs. 78.8 min/day (*p* = 0.179), 56.2 min/day vs. 54.6 min/day (*p* = 0.399) and 39.0 min/day vs. 40.6 min/day (*p* = 0.007), respectively.

Table 3 lists the multiple linear regression analysis for FIM-total, FIM-motor and FIM-cognitive. IS was independently associated with FIM-total (partial regression coefficient [B]: 5.378, 95% CI: 0.709–10.047) and FIM-cognitive (B: 1.792, 95% CI: 0.300–3.283), as well as age, FIM at admission, pre-stroke care needs and lower incidence of limb motor paralysis. However, IS was not independently associated with FIM-motor (B: 3.587, 95% CI: −0.194–7.368). After excluding seven tetraplegic (3%) participants, multiple linear regression analysis was performed on FIM-total, FIM-motor and FIM-cognitive using improvement in HG instead of IS. As a result, improvement in HG was independently associated with FIM-total (B: 5.685, 95% CI: 1.351–10.020) and FIM-motor (B: 5.007, 95% CI: 1.375–8.638). On the other hand, HG was not associated with FIM-cognitive (B: 0.657, 95% CI: −0.454–1.768).

In addition, multiple logistic regression analysis showed that age (odds ratio (OR), 0.945; 95% CI, 0.894–0.999) and FIM at admission (OR, 1.050; 95% CI, 1.020–1.070) were independently associated with the home discharge proportion of the stroke patients. The IS group tended to have higher odds of home discharge than the NIS group (OR, 2.560; 95% CI, 0.912–7.170). Furthermore, multiple logistic regression analysis of the home discharge proportion using improvement in HG instead of IS revealed that the improvement in HG was an independent explanatory factor (OR, 2.280; 95% CI, 1.010–5.140).

## 4. Discussion

We performed a cross-sectional study in stroke patients with sarcopenia in the convalescent rehabilitation ward. Three important findings were obtained with this study. First, the results demonstrated that 30% of stroke patients with sarcopenia at admission had recovered from sarcopenia at discharge. Second, the improvement in sarcopenia was independently associated with higher FIM-total and FIM-cognitive. Third, in this population, IS was tendentially associated with higher odds of home discharge than NIS.

In the present study, 30% of stroke patients with sarcopenia at admission recovered from sarcopenia at discharge. To the best of our knowledge, this is the first study to demonstrate the proportion of recovery from sarcopenia in patients with stroke. In addition, 65% of participants had increased muscle mass, while 70% of participants had increased muscle strength during hospitalization. In a previous study conducted in the convalescent rehabilitation ward, around 70% of stroke patients with sarcopenia showed increased muscle mass during hospitalization [34]. The result of the present study was in full agreement with this study. For patients with sarcopenia, resistance exercise, nutritional intervention (protein intake of 1.0–1.5 g/kg/day) and treatment of diseases related to sarcopenia are recommended [35,36,37]. The participants in this study had received appropriate nutritional therapy (with an average protein intake of about 1.1 g/kg/day) and a rehabilitation program with an average of around 180 min/day. The rehabilitation program as well as the nutritional management may have contributed in part to the improvement of sarcopenia in the patients, although specific intervention information was not collected.

The improvement in sarcopenia was independently associated with FIM-total and FIM-cognitive in patients with stroke undergoing convalescent rehabilitation. Although it is difficult to completely distinguish between the consequence of natural history of stroke and IS, we analyzed HG as a part of the criteria for sarcopenia to provide some evidence to IS. We excluded seven tetraplegic patients (3%) from the analysis and conducted multiple linear regression analysis for FIM-total, FIM-motor and FIM-cognitive using improvement in HG instead of IS. The results showed that the improvement in HG was significantly associated with FIM-total and FIM-motor. As the improvement in sarcopenia involves an increase in muscle mass and/or increase in HG, this result may indicate that improving sarcopenia is partially explained by an alternative pathway from the natural course of stroke recovery.

Moreover, it was reported that the presence of sarcopenia at the time of admission may lead to poor ADL outcomes in patients after stroke [12,15]. However, the results obtained in this study strongly suggest that, even if sarcopenia is present at the time of admission, the improvement of the condition may lead to better ADL outcomes. Therefore, we consider treatment of sarcopenia to be fundamental in order to achieve better ADL outcomes. Interventional studies and investigation of long-term outcomes are needed to further clarify this relationship. In addition, FIM-cognitive was associated with IS. This result is consistent with those of previous studies showing that sarcopenia may be a risk factor for cognitive decline [38,39]. The exact underlying mechanism has not yet been determined, but it has been reported that inflammation, myokines, etc., may partially explain the association between cognitive decline and sarcopenia [40,41]. However, FIM-motor was not associated with IS. One explanation for the association of FIM-motor and IS is its components (i.e., muscle strength and muscle mass). Muscle mass was reported to be a less predictive value for the functional outcome than muscle strength [42], and their composite (i.e., sarcopenia) might have less predictive value than muscle strength. Muscle strength was reported to be a better predictor of adverse outcomes than muscle mass [2,43]. Additionally, improvement in HG was an independent explanatory factor for FIM-motor in our study. This result suggests that muscle strength has more predictive value for motor function than muscle mass plus strength.

In addition, the IS group tendentially had a higher odds of home discharge than the NIS group, although the *p*-value obtained was above the significance level (OR, 2.560; 95% CI, 0.912–7.170). The relationship between sarcopenia and home discharge can be partially explained by the improvement in disability and inactivity. Physical disability and inactivity had been previously reported as factors related to returning home [44,45]. Similarly, sarcopenia is associated with functional decline and inactivity [5]. Moreover, the improvement in HG was an independent explanatory factor for the home discharge proportion (OR, 2.280; 95% CI, 1.010–5.140). Muscle strength is one of the evaluations of sarcopenia, and we believe that it can explain part of the relationship between sarcopenia and home discharge proportion. Therefore, an association between patients’ improvement from sarcopenia and recovery of ADL is plausible since our results showed a relationship between these variables.

Several limitations of this study should be mentioned. First, distinguishing whether sarcopenia was established after or before the onset of stroke could not be addressed. Second, interventions provided to the participants of this study, including rehabilitation therapy and nutritional care, were patient-specific and not standardized.

## 5. Conclusions

In conclusion, 30% of stroke patients with sarcopenia in the convalescent rehabilitation ward included in this study had recovered from sarcopenia at discharge. The improvement in the patients’ condition was independently associated with higher FIM-total and FIM-cognitive. In addition, the patients whose sarcopenic status improved had around 2.5 times higher odds of home discharge than those who did not. Recovery from sarcopenia based on consensus-based criteria may be associated with a better improvement in overall functional status. The results presented herein might offer new insights for outcome measures of intervention for stroke patients with sarcopenia.

## Figures and Tables

**Table 1:** Demographic characteristics of 227 post-stroke older patients with sarcopenia admitted to convalescent rehabilitation wards.

	Overall	Improvement in Sarcopenia	Non-Improvement in Sarcopenia	*p*-Value
Patients, *n*	227	69	158	
Age, years, mean (SD)	80.5 (7.7)	78.1 (6.8)	81.5 (7.9)	0.002 ^§^
Female sex, *n* (%)	125 (55)	41 (59)	84 (53)	0.468 ^‡^
Stroke subtype, *n* (%)				0.259 ^‡^
Ischemic stroke	164 (72)	46 (67)	118 (75)	
Hemorrhagic stroke	63 (28)	23 (33)	40 (25)	
CCI, score, median [IQR]	1 [0, 2]	0 [0, 2]	2 [0, 2]	0.017 ^†^
Days between onset and admission, days, median [IQR]	23 [19, 29]	23 [19, 31]	23 [19, 28]	0.643 ^†^
FIM, score, median [IQR]				
Total	68 [44, 86.5]	74 [56, 87]	63 [42.25, 85.75]	0.123 ^†^
Motor domain	45 [25.5, 60]	50 [32, 59]	42.5 [24, 60]	0.314 ^†^
Cognitive domain	21 [16, 28.5]	25 [19, 29]	20 [15, 27.75]	0.009 ^†^
Pre-stroke care needs *, *n* (%)	54 (24)	7 (10)	47 (30)	<0.001 ^‡^
Lower limb motor paralysis, *n* (%)				0.505 ^‡^
BRS I–IV	55 (24)	20 (29)	35 (22)	
BRSV–VI	123 (54)	36 (52)	87 (55)	
absence	49 (22)	13 (19)	36 (23)	
Height, cm, mean (SD)	153.9 (8.1)	155.1 (7.8)	153.4 (8.2)	0.139 ^§^
Body weight, kg, mean (SD)	49.9 (9.2)	52.4 (8.5)	48.7 (9.3)	0.005 ^§^
Body mass index, kg/m^2^, mean (SD)	21.0 (3.2)	21.7 (2.7)	20.7 (3.3)	0.019 ^§^
Skeletal muscle mass index, kg/m^2^, mean (SD)	5.3 (0.9)	5.6 (0.8)	5.1 (0.9)	<0.001 ^§^
Hand-grip strength, kg, mean (SD)	15.4 (6.3)	17.7 (6.6)	14.4 (5.9)	<0.001^§^
Tube feeding, *n* (%)	19 (8)	7 (10)	12 (8)	0.603 ^‡^
MUST, score, median [IQR]	1 [0, 2]	1 [0, 1]	1 [0, 2]	0.157 ^†^
Energy intake, kcal/kg/day, mean (SD)	27.5 (7.1)	26.4 (6.5)	28.0 (7.3)	0.126 ^§^
Protein intake, g/kg/day, mean (SD)	1.1 (0.3)	1.1 (0.3)	1.1 (0.3)	0.329 ^§^

CCI, Charlson Comorbidity Index; FIM, Functional Independence Measure; BRS, Brunnstrom recovery stage; MUST, Malnutrition Universal Screening Tool; IQR, interquartile range; SD, standard deviation. * Pre-stroke care needs confirmed by certification for public long-term care insurance. ^†^ Mann–Whitney U test. ^‡^ Chi-square test. ^§^ *t*-test.

**Table 2:** Outcome measures at discharge from the convalescent rehabilitation ward in 227 post-stroke older patients with sarcopenia.

	Overall	Improvement in Sarcopenia	Non-Improvement in Sarcopenia	*p*-Value
FIM, score, median [IQR]				
Total	104 [ 76.5, 117]	112 [91, 120]	101 [73, 116]	0.003 *
Motor domain	77 [54, 85]	81 [64, 87]	73.5 [50, 84]	0.009 *
Cognitive domain	28 [21, 32]	30 [26, 33]	26 [20, 31]	0.001 *
FIM gain, score, median [IQR]				
Total	28 [18.5, 38.5]	33 [26, 39]	24.5 [17, 36]	0.002 *
Motor domain	23 [15, 33]	28 [20, 33]	21 [12.25, 32]	0.002 *
Cognitive domain	4 [2, 7]	5 [2, 7]	4 [2, 6.75]	0.208 *
Length of stay, days, mean (SD)	101.7 (43.3)	105.7 (47.1)	99.9 (41.5)	0.351 ^‡^
Discharge outcome, *n* (%)				0.015 ^†^
Home	185 (82)	63 (91)	122 (77)	
Others	42 (19)	6 (8)	36 (23)	
Dairy rehabilitation dose, min/day, median [IQR]	176 [174, 178]	176 [174, 178]	176 [174, 178]	0.634 *
Skeletal muscle mass index, kg/m^2^, mean (SD)	5.5 (1.0)	6.1 (0.9)	5.3 (0.9)	<0.001 ^‡^
Hand-grip strength, kg, mean (SD)	17.5 (6.4)	21.2 (6.6)	15.9 (5.6)	<0.001 ^‡^

FIM, Functional Independence Measure; IQR, interquartile range; SD, standard deviation. * Mann–Whitney U test. ^†^ Chi-square test. ^‡^ *t*-test.

**Table 3:** Multiple linear regression analysis for FIM-total, FIM-motor and FIM-cognitive in 227 post-stroke older patients with sarcopenia. The association between each outcome and improvement in sarcopenia was determined.

	B	95% CI		*p*-Value
		Lower	Upper	
FIM-total	5.378	0.710	10.047	0.024
FIM-motor	3.587	–0.194	7.368	0.063
FIM-cognitive	1.792	0.300	3.283	0.019

Explanatory variables: age, sex, Charlson Comorbidity Index, days between onset to admission, Functional Independence Measure, pre-stroke care need, HG, skeletal muscle mass index, lower limb motor paralysis, stroke subtype, Malnutrition Universal Screening Tool score, tube feeding, energy intake and protein intake.

## Data Availability

The data presented in this study are available on request from the corresponding author when the ethics committee and the hospital where the study conducted permit.
